# Nutritional status of adolescents with cystic fibrosis treated at a reference center in the southeast region of Brazil

**DOI:** 10.1186/s13052-015-0159-x

**Published:** 2015-07-30

**Authors:** Ieda Regina Lopes Del Ciampo, Luiz Antonio Del Ciampo, Regina Sawamura, Laiane Renolfi de Oliveira, Maria Inez Machado Fernandes

**Affiliations:** Department of Medicine, Federal University of São Carlos, São Carlos, São Paulo Brazil; Department of Pediatrics, Ribeirão Preto School of Medicine, University of São Paulo, Ribeirão Preto, São Paulo Brazil; Ribeirão Preto School of Medicine, University of São Paulo, Ribeirão Preto, São Paulo Brazil

**Keywords:** Cystic fibrosis, Nutritional status, Adolescent, Puberty, Pancreatic insufficiency

## Abstract

**Background:**

Several factors can interfere with the full physical and emotional growth of adolescents, among them chronic diseases. The aim was to determine the nutritional status of adolescents and to associate it with puberty, pancreatic sufficiency, lung function and age range of Cystic Fibrosis (CF) diagnosis.

**Methods:**

An observational, cross-sectional, retrospective and analytical study was conducted using the data of medical records. Setting: Reference center in the northeastern region of the state of São Paulo – Brazil. Patients: All adolescents with CF attended in 2010 were included. Some variables included: pancreatic sufficiency (steatocrit >2 %), pancreatic enzymes replacement (yes/no), pubertal status-Tanner criteria (prepubertal: M1/G1, pubertal: M2/G2 to M4/G4, postpubertal: M5/G5), age at CF diagnosis (<2 and ≥2 years of age), Lung function, measured as a predicted forced expiratory volume in 1 s (FEV1). Main outcome measures Nutritional indicators: body mass index for age (BMI/A) and height for age (H/A) with z-score calculated with Anthro Plus software. Cut-off reference points: ≥ z-score −3 and < z-score −2 (thinness); z-score −2 and ≤ z-score-z +1 (normal weight); >z-score +1 (overweight or obesity), and z-score <−2 (low or very low H/A). The groups were compared by the Kruskal-Wallis test. Level of significance: *p* < 0.05.

**Results:**

Thirty adolescents. Median (min;max)  age: 14.4 (10.1;19.8) years. BMI/A and H/A z-score, respectively: early diagnosis of CF (−0.8; −1.1) or late diagnosis of CF (−0.5;-0.8); with pancreatic insufficiency (−0.7; −0.8) or without pancreatic insufficiency (−0.8; −0.5) and prepubertal (−0.8; −0.7) pubertal (−0.2; −1.5) or postpubertal (−0.7; -0.5). No significant difference (*p* > 0.05) was observed. Patients with and without pancreatic insufficiency, presented H/A borderline z-score (*p* = 0.05). Association between H/A and FEV1 was borderline (*p* = 0.05).

**Conclusions:**

Adolescents presented adequate nutritional status, although with slightly lower values than those of developed countries. FEV1 lower levels occurred more frequently in adolescents with low H/A.

## Background

Adolescence is a phase of life developing from 10 to 19 years which is characterized by an evolving period with marked body modifications and a high nutritional demand and culminating with biopsychosocial maturation [1]. Rapid linear growth, the pubertal spurt and sexual maturation occur during this period and are evaluated according to the different stages of the Tanner classification (M2 to M4 in girls and G2 to G4 in boys) [2]. This second decade of life is considered to be highly vulnerable since several factors can interfere with the full physical and emotional growth of adolescents, among them chronic diseases [3].

Cystic fibrosis (CF) is a lethal genetic disease of recessive autosomal inheritance commonly affecting Caucasian populations. CF is due to mutation of the Cystic Fibrosis Transmembrane Conductance Regulator gene which induces the production of thick and viscous secretions that obstruct the lungs, the pancreas and the bile duct. Among various therapeutic measures, this disease requires the use of pancreatic enzymes for patients with pancreatic insufficiency and a high-protein and high-calorie diet, which are associated with better pulmonary function and longer survival [4].

Over the last few years, due to advances in the early detection of the disease and to new treatments, the prognosis of CF patients has improved, with an increase in time and quality of life [5].

The objective of the present study was to determine the nutritional status of adolescents seen at a reference center and to associate it with the periods of puberty, with pancreatic sufficiency, with pulmonary function and with the age range of CF diagnosis.

## Methods

An observational, cross-sectional, retrospective and analytical study was conducted using the data of medical records of the Reference Center of Cystic Fibrosis of the University Hospital. The city of Ribeirão Preto has a Human Development Index of 0.800 and serves a population estimated at more than two million inhabitants residing in the northeastern region of the state of São Paulo. All adolescents treated in this service since its creation, with a diagnosis of CF based on clinical manifestations and two sweat chloride tests >60 mEq/L, and who had attended at least one medical visit in 2010 were included in the study. There were 136 patients attended in 2010 and 30 was adolescents. The sample size was 28 (IC 90 %), according with statcalc Epi-info seven, considering all patients attended and prevalence of 25 % of undernutrition in infants and adolescents with cystic fibrosis. However, we chose to work with convenience sample. As there were no losses, all 30 teenagers participated in the study. The variables studied were age (years), sex (M/F), reported color (white non-white), weight (g), height (cm), pancreatic sufficiency defined according to steatocrit >2 % and generating two subgroups, i.e., patients with pancreatic sufficiency (PS) and patients with pancreatic insufficiency (PI), use of pancreatic enzymes (yes/no), use of nutritional supplement (yes/no), chronic colonization to pseudomonas aeruginosa (yes/no), cystic fibrosis related diabetes - CFRD (yes/no), cystic fibrosis related hepatic disease - CFRHD. Lung function (forced expiratory volume in 1 s, measured as a percent of predicted normal values - FEV1). Biohumoral indirect parameters of patient’s nutritional state levels: haemoglobin g/dL (Hb), total protein g/dL (TP) and albumin g/dL. According to the Tanner criteria, pubertal status was categorized as prepubertal (M1/G1), pubertal (M2/G2 to M4/G4) and postpubertal (M5/G5) and the age range at CF diagnosis was defined as <2 and ≥2 years of age.

Anthropometric data such as weight and height were used to determine the nutritional status. The anthropometric indicators adopted were body mass index for age (BMI/A) and height for age (H/A) according to chronological age and sex. BMI was calculated as weight (kg) divided by height (m) squared. The z-score of the nutritional indicators was calculated with the Anthro Plus software using the cut-off reference points established by the WHO [6]: ≥ z-score −3 and < z-score −2 (thinness); z-score −2 and ≤ z-score-z +1 (normal weight); > z-score +1 (overweight or obesity), and z-score <−2 (low or very low H/A). The groups were compared by univariate analysis using the Kruskal-Wallis test of the EPI INFO 7 software, with the level of significance set at *p* ≤ 0.05.

The study was approved by the Research Ethics Committee of the University Hospital Ribeirão Preto.

## Results and discussion

The median (minimum; maximum) age of the 30 adolescent patients followed up at the service was 14.4 (10.1;19.8) years; 53.4 % were males, 86.7 % were white, 70 % had PI, and 23.3 % were prepubertal, as shown in Table 1.

**Table 1:** Characteristics of the 30 adolescents evaluated at the Reference Center of Cystic Fibrosis of HC - USP, 2010

Characteristics	Number	Percent
	Reported color	
White	26/30	86.7
Non-white	4/30	13.3
	Sex	
Male	16/30	53.4
Female	14/10	46.6
	Presence of pancreatic insufficiency	
No	9/30	30.0
Yes	21/30	70.0
	Use of pancreatic enzymes	
Yes	21/30	70
No	9/30	30
	Use of enzymes according to pancreatic sufficiency	
With pancreatic insufficiency	21/21	100
With pancreatic sufficiency	0/9	0
	Age at CF diagnosis	
<2 years	16/30	53.3
≥2 years	14/30	46.6
	Age at diagnosis of pancreatic insufficiency	
<2 years	16/21	76.2
≥2 years	5/21	23.8
	Pancreatic insufficiency in patients diagnosed before 2 years of age	
Yes	16/16	100
No	0/16	0
	Pubertal stage	
Prepubertal	7/30	23.3
Pubertal	11/30	36.7
Postpubertal	12/30	40.0

FEV1 medians (min; max)was 78.5 (41.0; 111) and 82.5 (45.0;98.0) considering that diagnosed before and after 2 years old, respectively (*p* > 0.05); 77.0 (45.0;102.0) and 85.0 (41.0;111.0) to the PI and PS, respectively (*p* > 0.05) and yet 82.0 (51.0;97.0); 86.0 (66.0;92.0) and 71.5 (41.0;111.0) to prepubertal, pubertal and postpubertal subgroups, respectively (*p* > 0.05).

FEV1 medians were 51.0 (51.0;51.0); 82.0 (41.0;111.0) e 88.0 (88.0;88.0) to thinness, normal weight and overweight or obesity (*p* > 0.05) and were 68.0 (45.0;88.0) and 84.0 (41.0;111.0) to low height and normal height, respectively (*p* = 0.05).

The FCDR was present in 13.3 % (4/30) and the FCDR in 26.7 % (8/30) of the adolescents.

BMI/A medians (min; max) was −0.64 (−3.7;+2.6) and −0.8 (−1.2;+0,7) to that without or with FCRD (*p* > 0.05) and BMI/A medians (min; max) was −0.75 (−3,7;+2.6) and −0.41 (−1.0;+0.74) to adolescents without or with FCDHR (*p* > 0.05), respectively. H/A means (SD) was −0.83 (−3.8;1.2) and −1.38 (−2.2;-0.18) to adolescents without or with FCDR (*p* > 0.05) and H/A means was −0.75 (−3.7;+1.2) and −1.0 (−2.8;+0.9) to adolescents without or with FCDHR, respectively (*p* > 0.05).

Considering all 30 adolescents, 73.3 % (22/30) were taking nutritional supplement. BMI/A medians (min; max), were 0.2 (−1.2;+2.6) and −0.72 (−3.7;+0.7) and HAZ means were −0.4 (−3.3;1.2) and −1.0 (−3.7;+0,9) to that adolescents without or taking nutritional supplement, respectively (*p* > 0.05).

Among prepubertal, pubertal and postpubertal subgroups it were observed the medians (min;max) of Hb 13.4 (11.9;14.7), 13.7 (11.7;15.6) and 13.6 (10.3;16.5); TP 7.1 (6.7;8.0), 6.8 (6.3;7.9) and 7.5 (6.1;7.8) and albumin 4.3 (3.9;4.7), 4.3 (3.6;4.6) and 4.1 (3.3;4.6); respectively (*p* > 0,05).

Table 2 presents the variables of the study according to age, BMI/A and H/A. No significant difference in mean age, BMI/A or H/A was observed between adolescents with and without PI. Also, no significant difference was observed regarding age at CF diagnosis or stages of pubertal development.

**Table 2:** Distribution of the variables according to age, BMI/A and H/A of the 30 adolescents evaluated at the Reference Center of Cystic Fibrosis of HC - FMRP - USP, 2010

Variables		Median	min; max	*p* value
	Age (years*)*			
Pancreatic sufficiency	Pancreatic Sufficient	14.2	10.77; 14.7	>0.05
	Pancreatic Insufficient	14.5	10.1; 19.8	
Pubertal stage	Prepubertal	10.7	10.1; 14.7	>0.05
	Pubertal	12.9	10.9; 19.0	
	Postpubertal	17.4	14.2; 19.8	
Age at Cystic Fibrosis diagnosis	<2 years	14.6	10.3; 19.8	>0.05
	≥2 years	12.8	10.1; 19.4	
	*BMI/A* ^*a*^ *(z score)*			
Pancreatic	Pancreatic Sufficient	−0.8	−1.5; 1.7	>0.05
Sufficiency	Pancreatic Insufficient	−0.7	−3.7; 2.6	
Pubertal stage	Prepubertal	−0.8	−3.7; 0.2	>0.5
	Pubertal	−0.2	−1.0; 2.6	
	Postpubertal	−0.7	−2.4; 2.7	
Age at Cystic Fibrosis diagnosis	<2 years	−0.8	−2.4; 1.7	>0.05
	≥2 years	−0.5	−3.7; 2.3	
	*H/A* ^*b*^ *(z score)*			
Pancreatic	Pancreatic Sufficient	−0.5	−2.9; 0.3	=0.05
Sufficiency	Pancreatic Insufficient	−0.8	−3.7; 1.3	
Pubertal stage	Prepubertal	−0.7	−1.8; −0.2	>0.05
	Pubertal	−1.5	−3.3; 1.2	
	Postpubertal	−0.5	3.7; 0.9	
Age at Cystic Fibrosis diagnosis	Before 2 years	−1.1	−3.7; 0.3	>0.05
	After 2 years	−0.8	−3.3; 1.2	

^a^ *BMI/A* Body mass index of age

^b^ *H/A* height for age

The results observed in this study allow the discussion, then, of some aspects. BMI/A has been recently adopted as a parameter for the surveillance of nutritional status in CF patients older than 2 years, and values above the 50th percentile are known to correlate with better pulmonary function [7]. A BMI/A < 15th percentile (p15) has been indicated as the ideal cut-off point for the definition of malnutrition in CF patients and the Cystic Fibrosis Foundation has recommended the BMI/A > 50th percentile as the goal to be reached for the nutritional status of these individuals [8]. Although without the rigor of the parameters adopted for consensus [9], in the present study the adolescents showed a BMI/A z-score of −0.6 (−3.7; 2.6) which corresponds to adequate nutritional status according to the parameters of the World Health Organization [6]. A study conducted in Germany on CF patients aged 4 to 18 years detected a BMI/A z-score of −0.1 [10].

The BMI/A z-score was similar for the present adolescents with and without pancreatic insufficiency (−0.7 and −0.8; respectively), whose nutritional status, therefore, was of adequate level according to WHO standards. These findings were close to those detected in an Italian study on patients younger than 18 years whose BMI/A z-scores were 0.2 and −0.3 for PS and PI, respectively [11]. It should also be pointed out that all the present adolescents with PI took pancreatic enzymes, which are extremely important for the maintenance of nutritional status in such patients [12].

It is known that, the sooner the diagnosis of CF is established, the more serious the symptoms and the shorter the survival [13]. However, in the present study, BMI/A z-scores did not differ significantly between an early or late diagnosis of CF, being −0.8 and −0.5, respectively. Although the present patients were not routinely genotyped, the results obtained agree with those published by investigators from developed countries, who reported that some patients with the classical manifestations of CF experience a mild disease course and that, in addition, an important proportion of patents with the severe and common delta F 508 mutation survive to 40 years of age with good maintenance of weight and pulmonary function [14].

Also concerning the nutritional status, the present group of prepubertal adolescents obtained the lowest BMI/A z-score (−0.8). The pubertal and postpubertal adolescents had z-scores of −0.2 and −0.7, respectively, all of them being considered nutritionally adequate. Because in this phase of life complications such as hepatic diseases, diabetes related to CF or even worsening of pulmonary function may further aggravate the nutritional status, more complications may have occurred in the prepubertal group, since these factors were not controlled due to the limited sample size [15, 16].

However, the better BMI/A z-scores detected in the pubertal and postpubertal groups may have been due not only to a satisfactory weight gain, but also to the lack of a height gain adequate for age, since this indicator underestimates overweight and underdiagnoses short stature in children and adolescents [17–19], a fact that underscores the importance of evaluating the H/A indicator.

The H/A ratio expresses linear growth and is considered to be the nutritional indicator that best points out the cumulative effect of adverse situations and the most sensitive one for the determination of quality of life in a population [20]. A low H/A (<5th percentile) has been recognized as a prognostic indicator of survival for CF patients [21]. Among CF individuals, adolescence is a period of high risk for delayed pubertal growth spurt [22] and impaired final height [23, 24], which appears to be mitigated by a better nutritional status at 7 years of age [18].

The H/A z-score of −0.8 (−3.7; 1.2) detected in the present patients is within adequate levels according to WHO standards, without corresponding to a low H/A, which characterizes worse survival among CF patients. Similar results were obtained by Groeneweg et al. in Germany [10].

No significant difference in H/A z-score was observed between patients with and without PI, although the borderline value of *p* = 0.05 may indicate impaired height in PI patients compared to PS patients. Indeed, the nutritional status of individuals with PS could be expected to be better than that of individuals with PI, a result also observed in a study conducted in Italy, in which significant differences in z-score (*p* < 0.03) were detected between PS and PI subjects [11].

Regarding the different stages of adolescence, the H/A z-scores did not differ significantly (*p* > 0.05) between prepubertal (−0.7), pubertal (−1.5) and postpubertal (−0.5) adolescents. Similarly H/A z-scores did not differ significantly (*p* > 0.05) between the groups diagnosed with CF before (−1.0) or after two years of age (−1.3). The different stages of adolescence and the early or late diagnosis of CF were not sufficient to differentiate the nutritional status of the various adolescent subgroups studied, with adequate H/A levels being observed in all of them according to the WHO.

The pulmonary function between BMI/A subgroups was not significant (*p* > 0.05), although 51.0 % adolescents with thinness presented lower FEV1 median. In turn, lower FEV1 means observed in 65.5 % adolescents with low height revealed that pulmonary function between H/A subgroups was significant (*p* = 0.05). Other researchers observed strong association between FEV1 and weight-for-height (ref. [25]).

The retrospective collection of anthropometric data is one of the limitations of the present study, although weight and height were measured by personnel trained by specialists. Adequate means of Hb, PT and albumin, corroborates the means of the anthropometric nutritional indicators observed in this study. The absence of genotyping and the non-exclusion of patients with major complications such as liver disease and diabetes related to CF are also limitations, which, however, did not overestimate the values observed.

## Conclusions

Although the need for a more rigorous nutritional assessment of individuals with CF is recognized, the adolescents studied here showed adequate nutritional status related to BMI/A and H/A, although with slightly lower values than those of developed countries according to WHO standards.

Parameters regarding an early or late diagnosis of CF and the pubertal stage of these adolescents did not reveal differences in nutritional status when BMI/A or H/A were considered.

BMI/A values did not differ in terms of status of pancreatic sufficiency, whereas H/A values were borderline for the determination of height impairment in adolescents with PI compared to adolescents without PI. FEV1 lower levels occurred more frequently in adolescents with low H/A.
